# Molecular Mechanisms of Neuroinflammation in ME/CFS and Long COVID to Sustain Disease and Promote Relapses

**DOI:** 10.3389/fneur.2022.877772

**Published:** 2022-05-25

**Authors:** Warren Tate, Max Walker, Eiren Sweetman, Amber Helliwell, Katie Peppercorn, Christina Edgar, Anna Blair, Aniruddha Chatterjee

**Affiliations:** ^1^Department of Biochemistry, University of Otago, Dunedin, New Zealand; ^2^Brain Health Research Centre, University of Otago, Dunedin, New Zealand; ^3^School of Biological Sciences, Victoria University of Wellington, Wellington, New Zealand; ^4^Graduate School of Health, University of Technology Sydney, Sydney, NSW, Australia; ^5^Department of Pathology, University of Otago, Dunedin, New Zealand

**Keywords:** ME/CFS, Long COVID, systemic inflammation, neuroinflammation, disease persistence, relapse

## Abstract

Myalgic Encephalomyelitis/Chronic Fatigue Syndrome (ME/CFS) is a disease now well-documented as having arisen commonly from a viral infection, but also from other external stressors, like exposure to agricultural chemicals, other types of infection, surgery, or other severe stress events. Research has shown these events produce a systemic molecular inflammatory response and chronic immune activation and dysregulation. What has been more difficult to establish is the hierarchy of the physiological responses that give rise to the myriad of symptoms that ME/CFS patients experience, and why they do not resolve and are generally life-long. The severity of the symptoms frequently fluctuates through relapse recovery periods, with brain-centered symptoms of neuroinflammation, loss of homeostatic control, “brain fog” affecting cognitive ability, lack of refreshing sleep, and poor response to even small stresses. How these brain effects develop with ME/CFS from the initiating external effector, whether virus or other cause, is poorly understood and that is what our paper aims to address. We propose the hypothesis that following the initial stressor event, the subsequent systemic pathology moves to the brain *via* neurovascular pathways or through a dysfunctional blood-brain barrier (BBB), resulting in chronic neuroinflammation and leading to a sustained illness with chronic relapse recovery cycles. Signaling through recognized pathways from the brain back to body physiology is likely part of the process by which the illness cycle in the peripheral system is sustained and why healing does not occur. By contrast, Long COVID (Post-COVID-19 condition) is a very recent ME/CFS-like illness arising from the single pandemic virus, SARS-CoV-2. We believe the ME/CFS-like ongoing effects of Long COVID are arising by very similar mechanisms involving neuroinflammation, but likely with some unique signaling, resulting from the pathology of the initial SARS-CoV-2 infection. The fact that there are very similar symptoms in both ongoing diseases, despite the diversity in the nature of the initial stressors, supports the concept of a similar dysfunctional CNS component common to both.

## Introduction

Since the 1930s, when documentation of post-viral fatigue illnesses first occurred, there have been 75 reported outbreaks of probable viral origin that have led to long-term diseases that have the profile of Myalgic Encephalomyelitis/Chronic Fatigue syndrome (ME/CFS). Of these, the most publicized have been Los Angeles (1934) (1), Akureyri, Northern Iceland (1946–1948) (2), the Royal Free Hospital in London (1955) (3), Incline Village, Nevada (1984) (4), and in New Zealand, Tapanui (1984) (5). That outbreak has become known in New Zealand's “health folklore” as “Tapanui flu.” The name ME originated from the London outbreak, and the name CFS from that in Incline Village, Nevada. These were outbreaks where the initial infectious agent affected a relatively modest number of people in the hundreds in stark contrast to the current pandemic where nearly 500 million to date have been affected. The condition in recent times generally has become referred to as ME/CFS, the combined name derived from the European and American outbreaks. Connections among these disease outbreaks are discussed more comprehensively in Van Elzakker et al. (6). Despite their frequency and documented occurrence over a relatively long period of time, little research investment into their understanding has occurred, perhaps because the outbreaks were isolated geographically, involved relatively few people each time and, despite being highly debilitating and lifelong, were dwarfed in importance by the more common abundant non-communicable diseases. The underlying biology of ME/CFS has not been well-understood, with blood screens using standard laboratory tests falling within the normal range. Although these affected patients were obviously ill, they were given a “clean bill of health” often with no medical incentive to dig deeper into the condition.

The worldwide prevalence of ME/CFS has been reported as between 0.4 and 2.6% among countries and cultures meaning it is ~10-fold more prevalent than other fatigue illnesses like multiple sclerosis (7). The lack of conclusive biomarkers for a diagnostic laboratory test and the ill-defined pathophysiology have hindered an understanding of the disease. There have been up to 20 different clinical case definitions proposed, and while the Fukuda 1994 diagnostic criterion has been commonly used by researchers and clinicians (8), more recent criteria like the Canadian Consensus Criteria (2003) developed by an international ME panel of experts are now favored (9). These criteria focused on core symptoms of post-exertional malaise, fatigue, sleep dysfunction, and cognitive dysfunction. Additionally neurological and autonomic/neuroendrocrine/immune symptom groups were included (9). These were further refined in 2011 to include emphasis on inflammation, neuropathology and energy impairments (10). Starting in the mid 1980's, the number of studies has accelerated to reveal biological dysfunctions in many physiological systems. The known features of the pathogenesis of ME/CFS have recently been elegantly summarized and compared with what was known in the early stages of the post-COVID-19 syndrome (11). They speculated the pathogenesis of this illness may be similar to ME/CFS.

Ironically, not long before the current viral outbreak of SARS-CoV-2, wider interest and investment in ME/CFS research began to increase, following publication of the US National Academy of Science's Institute of Medicine report in 2015 (12) that affirmed ME/CFS was a serious disease and had not received proper acknowledgment and recognition from the medical and research communities. A recent report from clinical experts of ME/CFS diagnosis and management highlighted the lack of knowledge of many clinicians to “appropriately diagnose or manage ME/CFS” and these experts provided recommendations for health care providers from their decades of clinical experience, and from recent scientific progress in understanding the disease. They report a staggering 90% of those with the disease may be undiagnosed or misdiagnosed (13).

The current pandemic has changed that scenario dramatically as the global viral infection of SARS-CoV-2 has now affected huge numbers of people across all countries (14) and it has spawned a serious ongoing post-viral syndrome, generally known as Long COVID, affecting up to 30% of those infected (15). It has now been renamed by the World Health Organization (WHO) as “Post-COVID-19 condition” (PCC) (16) together with development of an iteratively-derived clinical case definition, but that may encompass only a large subgroup of those with Long COVID. Initially, classification of Long COVID following acute SARS-CoV-2 infection would have included people with ongoing specific injury to the lungs, heart, kidney, and brain, as well a classic post-viral fatigue condition. It was proposed also one subgroup might be suffering from post-traumatic stress disorder arising from their hospital treatment. Nevertheless, the implication was that a considerable proportion of Long COVID sufferers had a very similar pathogenesis to that of ME/CFS (11). The ongoing pandemic would likely already have added 100 million cases of a post-viral fatigue syndrome (17) to the estimated 20 million cases of ongoing ME/CFS globally. With the long-term prognosis still not clear, it will add a highly significant new health burden. The new WHO clinical case definition for PCC is very similar to the more recently refined versions of the generic clinical case definition for ME/CFS indeed suggesting the fatigue syndromes are very closely related (18). One difference was that the condition had to persist for 3 months before confirmation of diagnosis, whereas for ME/CFS it is 6 months. It is this large subgroup of Long COVID patients that the hypothesis and discussion in this paper encompasses.

Since this ME/CFS-like illness of Long COVID has brought the post-viral syndromes very much into the eyes of the medical community as well as the public, there is hope that ME/CFS will no longer remain a “hidden” or “forgotten” illness. We are surely on the cusp of a new era of research that will bring welcome new understanding to these post-viral fatigue illnesses with hope of discovering new ways of ameliorating the debilitating symptoms that so restrict the lives and functionality of the affected patients. As ME/CFS does not seem to be neurodegenerative, a way to reverse both ME/CFS and Long COVID and allow the body to undergo the normal healing process may be possible. However, Komaroff has raised the possibility that an ancient transient biological response to injury or potential injury has been damaged in ME/CFS so it cannot be easily reversed as would normally occur (19).

When the body's immune system is activated, as with viral illnesses or stress responses, inflammatory cells are mobilized, but the intensity and duration is the determinant of whether the immune signaling is supportive of healing or is destructive to normal physiology (20). Brief controlled inflammatory responses are beneficial to remove a threat but if prolonged, as in ME/CFS, inflammatory cells and cytokines continue to respond inappropriately despite there no longer being any outside danger (21). Chronic inflammation can cause a range of symptoms from pain to fatigue, and fever to rashes. Inflammation is well-established as being associated with a range of common diseases like Alzheimer's disease, multiple sclerosis, cancer, heart disease, diabetes, and rheumatoid arthritis (22). A consequence of chronic inflammation in ME/CFS is a breakdown in homeostasis, and neuroinflammation helps to maintain the systemic chronic inflammation and immune dysfunction. Inflammation in simple terms can be described as a grouping of our immune cells in the extracellular area specific to a site of damage or infection (21), for example in the lungs with SARS-CoV-2 infection, although systemic inflammation is more widespread. The brain and other components of the central nervous system (CNS) form an enclosed compartment in the healthy state. It has its own immune response machinery that when activated can lead to neuroinflammation as a protective response. Just as in the systemic system, excessive or chronic neuroinflammation can be damaging. There is growing evidence that neuroinflammation is an important component of ME/CFS and, likely as well, of Long COVID as described below (23). Microglia, the immune cells of the CNS, when activated, are spread through the brain and spinal cord, and may account for ~15% of its cells (24). They act as the first responders to provide an active defense in the CNS against infectious agents, foreign bodies, or abnormal structures like amyloid plaques in Alzheimer's disease, and α-synuclein aggregates in Parkinson's disease. They can control damaged or excessive numbers of neurons as part of normal physiology and provide factors to facilitate remodeling of synapses (25). However, chronically activated microglia promote inflammatory functions that lead to neurological dysfunction (26), characteristic of that seen in ME/CFS. Mackay and Tate proposed neuroinflammation in the brain is fundamental for both sustaining ME/CFS and for facilitating the frequent more severe relapses of the illness in response to environmental, physical, emotional, or psychological stresses (27). With sensitivity to stress so dominant in ME/CFS, this hypothesis proposed the cluster of neurons within the paraventricular nucleus (PVN) responsible for processing stress are dysfunctional because of the neuroinflammation. This was proposed as the key to sustaining the illness irrespective of the unique characteristics of the initial stressor event, whether it be virus, other infectious agent, a toxic chemical, surgery or simply a severe life event. While brain imaging studies are still in their infancy to be able to give precise strong supporting evidence, the studies so far as described below have provided support for the concept. Mackay has extended these concepts in a model to explain the long-term effects of Long COVID (28).

### The Hypothesis

What has not been clear in ME/CFS and now in Long COVID, and what we are trying to address here is the connection between the inflammatory/immune dysregulation within the peripheral system caused by the initial infection or stress event, and the development of the chronic stress response and activation of the CNS's own inflammatory/immunological response that then leads to the sustaining of the ME/CFS and frequent relapses of the illness. Molecular and cellular and signaling mechanisms are likely to be mediating this connection.

**Our hypothesis**: Following activation of a systemic immune/inflammatory response to an infection or severe stress event, abnormal transport of signals or molecules into the CNS occurs through neurovascular pathways or a disrupted BBB. If the initial stressor is not resolved this leads to fluctuating chronic neuroinflammation that sustains and controls the complex neurological symptoms of ME/CFS and Long COVID and facilitates frequent more serious relapses in response to life stress, as evidenced from a comprehensive disruption to the cellular molecular biology and body's physiological pathways.

**Figure 1:**
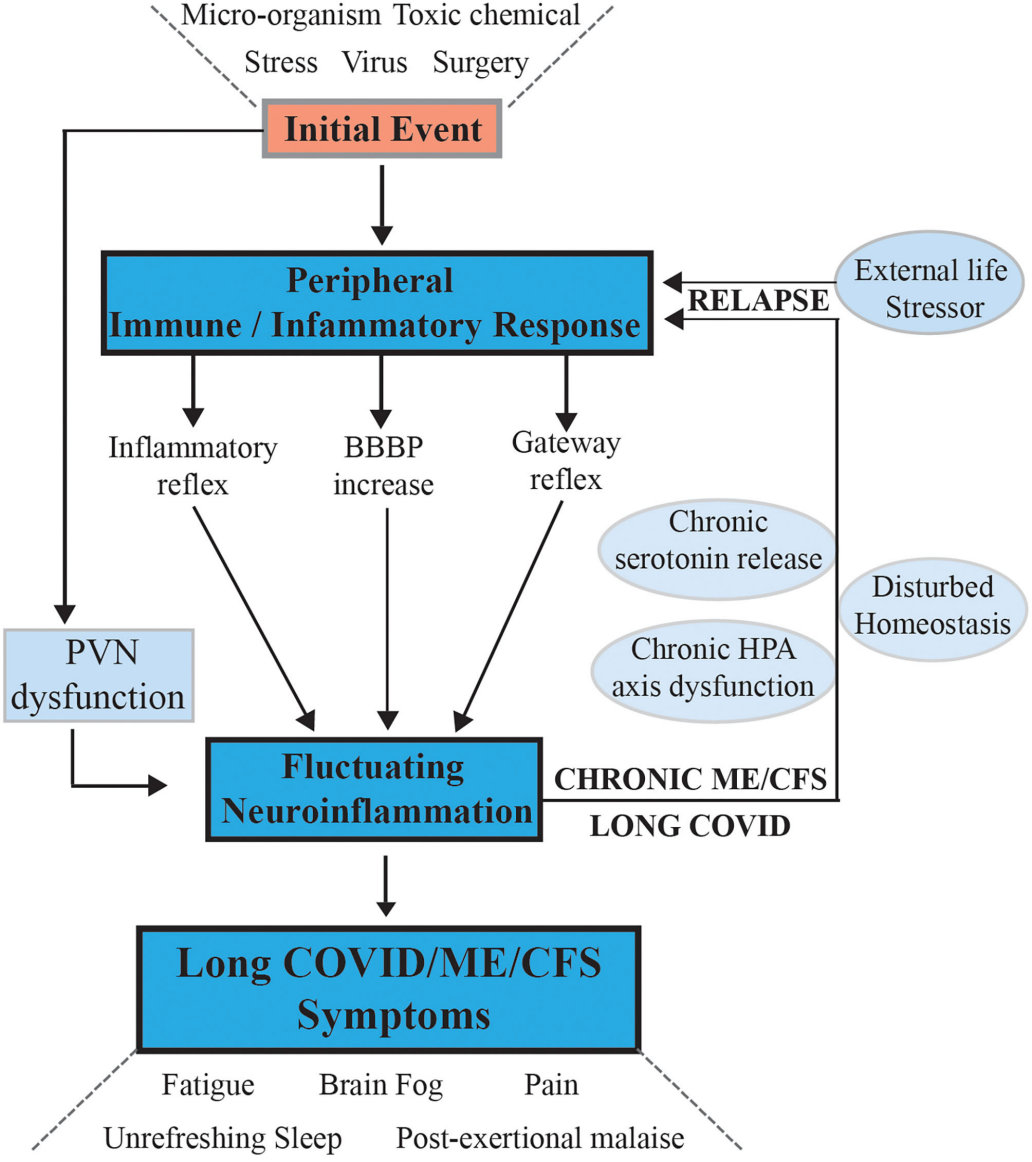
Hypothesis and model for onset of ME/CFS and its progression to a chronic sustained illness with relapse/partial recovery phases: signaling pathways between the CNS and the periphery that maintain the illness. Following an initial external stressor event systemic immune/inflammatory responses are activated, these are communicated to the CNS *via* inflammatory, and gateway reflexes and possibly an increase in permeability of the BBB. Neuroinflammation is activated affecting the stress center within the PVN of the hypothalamus and leads to a wide range of neurological symptoms that feedback to the periphery *via* disturbance of homeostasis and the stress activated HPA axis that becomes dysfunctional with chronic activation. The systemic physiology and molecular homeostasis are then chronically affected through important cellular functions like mitochondrial energy production metabolic activity and a continuation of immune/inflammatory reactions. External life stressors that feed into a disturbed PVN not only maintain the ME/CFS but also act to precipitate relapses.

For the initial part of our hypothesis, as shown in Figure 1 we propose that the systemic inflammatory state is initially transferred to the CNS if the immune inflammatory response of the peripheral system does not subside rapidly to a normal state as happens in most people exposed to viral infections or transient life stresses. Instead, if it becomes chronic and dysregulated as is the case in ME/CFS, then atypical signaling to the brain and central nervous system occurs to chronically activate and sustain the specific components of the microglial mediated immunological/inflammatory response that results in chronic neuroinflammation.

Questions relevant to the hypothesis that must be further developed are (i) through what mechanism(s) does the neuroinflammation arise, and how is it maintained in ME/CFS and (ii) how does this manifest the myriad of symptoms seen in ME/CFS? Our hypothesis proposes that abnormal signaling or transport of molecules/cells occurs through one or both of neurovascular pathways and/or a dysfunctional blood brain barrier (BBB). That is, the normally separate and contained brain/CNS compartment in the healthy person becomes more porous. It implies initial systemic inflammation caused by a viral/bacterial infection or stress event might lead to, for example, inflammatory signals or immune cells/molecules migrating into the brain. If it persists, strong signals are sent along peripheral afferent neurons culminating in the neuroinflammatory response. What results are the neurological symptoms characteristic of ME/CFS. The CNS through the hypothalamus/paraventricular nucleus and the brain stem can then signal back to the peripheral system to modulate abnormally much of the body's homeostatic state and physiology through well-established pathways (27, 28). The resulting symptoms and the neurologically driven “sickness response” for the ME/CFS patient would persist, preventing healing and a return to the pre- infectious/stress-related state. This may relate to the neuronal based signaling of perceived injury or danger (19, 29), which persists and leads to a hibernation-type state with lowered metabolism (30, 31) and altered molecular biology in ME/CFS (32–34). This “perception of danger” response may be damaged by the strong immune/inflammatory response in ME/CFS (a “cytokine storm”) and thereby not easily reversed. A cycling of molecular “danger signaling” signals between the systemic innate immune system and brain's innate immune system may then be set up and persist. This might occur for example, from damaged mitochondria acting as a signaling organelle, for example with leakage of ATP and subsequent purinergic signaling to the microglia (35). A dysfunctional physiology, with a dysregulated cellular molecular biology also becoming a critical facet, results in a disruption of the normal carefully balanced homeostasis. The inability of the brain to manage stress in ME/CFS and Long COVID patients *via* the stress center in the paraventricular nucleus (PVN) of the hypothalamus means stress would be a constant fuel stoking the immune response in the CNS to create relapse and partial recovery cycles (27, 28).

The PVN is now believed to be one of the most important autonomic control centers of the brain and it contains specific neurons that control stress and functions like metabolism and immune response, disturbed in ME/CFS, as well as autonomic functions of the gastrointestinal system, and cardiovascular system that are also affected. There are axonal connections from the PVN to the brain stem and spinal cord involved in controlling these autonomic outputs (36). The PVN is responsible for modulating the body's response to day to day stressors *via* the parvocellular neurosecretory cells that secrete corticotropin-releasing hormone (CRH), which is involved in autonomic and homeostatic regulation (37). Release of CRH can lead to activation of the brain's immune cells, microglia, mast cells and T cells, which in turn lead to neuroinflammation through the release of inflammatory mediators (38). The PVN can also alter sympathetic nerve activity in response to humoral factors in circulation. These can be detected through circumventricular organs that lack a blood-brain barrier (BBB) such as the median eminence, subfornical organ and organum vasculosum lamina terminalis, which suggest there is a pathway through which systemic inflammatory molecules can elicit a response from an important stress response center in the brain (39, 40). Furthermore, it has been shown that microglia in the PVN are activated by psychosocial stressors indicated by raised levels of Iba-1 and are also activated as a result of hypertension (39, 41). Hatziagelaki et al. has also suggested this abnormal state of homeostasis in ME/CFS could be caused by inflammation in the hypothalamus (42).

## Evidence of an Ongoing Systemic Peripheral Immune/Inflammatory System Dysfunction in ME/CFS

Studies have shown ongoing immune dysfunction in ME/CFS patients, both in the blood borne immune cells (43) and in the immune regulator molecules (cytokines) that they export (44, 45). It is commonly stated the ME/CFS patients' immune systems are chronically activated or in “overdrive” (46). These studies above have shown cytokine signatures characteristic of the severity of the disease that are mainly proinflammatory. Montoya et al. examined the human system for markers of inflammation and found 13 of 17 cytokines that correlated with the severity of ME/CFS were proinflammatory and thereby were likely to be contributing significantly to the symptoms (44). A distinct cytokine inflammatory signature associated with early disease has been reported in ME/CFS (47). Early ME/CFS cases had a prominent activation of both pro- and anti-inflammatory cytokines as well as dissociation of intercytokine regulatory networks, signatures that were not found at later stages of the illness. Mandarano et al. investigated immune cell metabolism in ME/CFS, analyzing glycolysis and mitochondrial respiration in resting and activated T cells, along with markers related to cellular metabolism and plasma cytokines. In ME/CFS both CD4^+^ and CD8^+^ T cells had reduced glycolysis at rest, whereas CD8^+^ T cells also had reduced glycolysis following activation. Patients with ME/CFS had significant correlations between measures of T cell metabolism and plasma cytokine abundance that differed from correlations seen in healthy control subjects (45).

Our own research studies have examined immune cell molecular biology for ongoing dysfunction in patients in the chronic phase of ME/CFS with an average time since disease onset of 10 years. It has used the concept of “personal-omics” (48) to determine dysregulated molecular pathways and dysfunctional physiology from analysis of many thousands of molecules in different molecular classes and DNA methylation sites across the genome for changes in the epigenetic code (32–34). A pilot study is now underway to compare age/gender matched ME/CFS patients, Long COVID patients, and healthy controls to determine how closely the molecular changes in two post-viral fatigue illnesses correlate (Tate—unpublished). An investigation of a potential molecular diagnostic test for ME/CFS, based on measuring an activated stress kinase that is characteristic of a chronically activated immune system, has shown promising results even in patients in the ongoing chronic state of ME/CFS (7). All these studies support the concept of a peripheral cell immune system in “overdrive” (47). Collectively and consistently the results have clearly shown evidence of disturbed immune function and regulation, strong association with inflammatory responses, as well as dysfunctional energy production, a lowered rate of metabolism, impaired circadian clock function, and they have suggested there are neurological defects (32–34). Just as there have been a documentation of disturbed metabolic homeostasis (30), the proteome and methylome studies suggest molecular homeostasis is significantly disturbed as well. Critically, in mitochondria this can lead to compromised energy production that may have repercussions throughout all body systems and an ability to provide extra energy to manage stress. Our most recent study has followed individual patients through a “relapse/recovery cycle” and has indicated there is a stronger peripheral immune dysfunction and greater inflammatory response during relapse that we infer arises from increased neuroinflammation and, from the altered DNA methylation profiles, changes in the expression of critical genes that would likely mediate this peripheral response in the immune cells (49). These studies from our group are summarized in the following Sections: Changes in the Transcriptome of Immune Cells in ME/CFS Patients, Differentially Expressed Proteins in the Immune Cells of ME/CFS Patients Analyzed by Sequential Window All Theoretical Spectra Mass Spectrometry MS (SWATH-MS), and A Comparison Between the ME/CFS Patient Cohort and the Healthy Controls Showed Changes in the DNA Methylation Profiles That Reflect Systemic Dysfunctions That Become More Pronounced During a Relapse.

### Changes in the Transcriptome of Immune Cells in ME/CFS Patients

The analysis of functional association networks of proteins encoded by differentially expressed gene transcripts (*n* = 33, *P* < 0.01) among the 13,000 transcripts analyzed in ME/CFS patients compared with healthy controls were performed using the STRING portal (http://string-db.org, version 11). The analysis demonstrated a significant level of network enrichment indicating the encoded proteins are biologically connected (32). Strong functional associations were observed between “inflammatory” transcripts, specifically the inflammatory cytokine, *IL8*, and two anti-inflammatory inhibitory transcripts *NFKBIA, TNFAIP3*. Other differentially expressed transcripts *JUN, NAMPT, CREM, PMAIP1, PPP1R15A, RBBP6, UBE2D3, SOCS3, RIPK2*, and *ZC3H12A* reinforce that there is significant immune and inflammatory over-activation and dysregulation occurring in the ongoing pathology of ME/CFS (32).

Ingenuity Pathway Analysis (IPA) implicated inflammation in ME/CFS. IPA was carried out on a differentially expressed transcriptome dataset of 165 with significance set at *P* < 0.05 to increase the pool available for analysis. Functionally interlinked canonical pathways were directly related to inflammatory signaling and the stress response, supporting the functional network analysis. Gene networks related to oxidative stress, inflammation, and nervous tissue damage were also identified.

**Figure 2:**
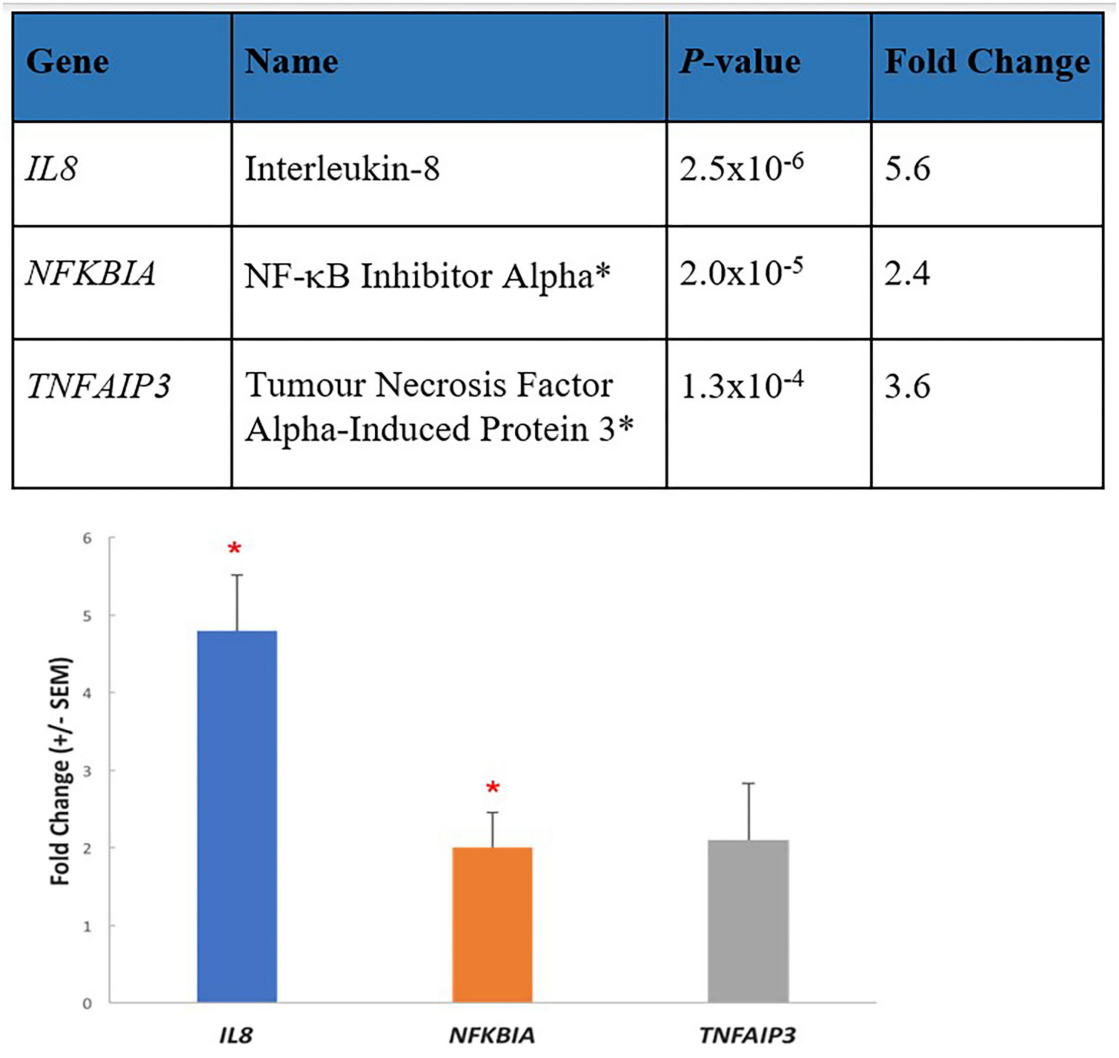
Three key elevated transcripts in immune cells from ME/CFS patients are involved in inflammation. The most enhanced transcripts from RNA-seq data (*p*-value 0.01/fold change 1.5) in PBMCs from 10 ME/CFS patients compared with 10 age/gender matched healthy controls shown in the upper part of the figure were Interleukin 8 (IL8) a proinflammatory cytokine, and two inhibitory transcripts, NF-κB inhibitor alpha (*NIFKBIA*), and tumor necrosis factor alpha-induced protein 3 (*TNFAIP3*). These were validated by determining the mean fold changes by RT-qPCR (±SEM) between the patient group and the healthy control group, and similar fold changes were determined. *p < 0.01.

The three top upregulated genes in our study, *IL8, NFKBIA*, and *TNFAIP3*, validated by qPCR (see Figure 2), are early-responders to TNF-induced NF-κB activation (50). Chronic inflammation is amplified by the NF-κB signaling pathway (51). The encoded proteins A20 (*TNFAIP3*) and IκBα (*NFKBIA*) are key components of the two main negative feedback loops of NF-κB-driven transcription (52). TNFα is a potent inducer of IL-8 secretion through a transcriptional mechanism regulated by NF-κB. Increases in IL-8 and TNFα have both been identified in several ME/CFS studies (6). We can conclude that the increase in expression of these three gene transcripts in the ME/CFS group implies an underlying biological counter-attempt to control unwanted excess activity of NF-κB and inflammation in ME/CFS, driven by TNFα. TNFα-driven inflammation and inflammatory markers such as *IL8* are also indicative of neuroinflammation (53). An inflammation amplifier as discussed below is driven by a simultaneous activation of NF-κB and STATs in non-immune cells, causing the production of a burst of inflammatory chemokines to open a gateway to the CNS, a key feature of our hypothesis.

Functional network analysis had shown an increased cellular stress response and positive regulation of apoptotic processes in the ME/CFS group. IPA highlighted the production of nitric oxide (NO) and reactive oxygen species (ROS) in macrophages. NO and ROS production, stimulated by TNFα and NF-κB, play a central role in the control of infections (54). ROS production is also attributed to activated NADPH oxidase, a component of the mitochondrial electron transport chain (55). Mitochondria densely populate cells in the CNS, providing essential energy for neurons and thereby influencing synaptic plasticity (56). Oxidative stress, caused by increased ROS in mitochondria, can lead to apoptosis and cause mitochondrial DNA damage that could affect neurotransmission and Ca^2+^ homeostasis (57).

IPA identified the NF-B activation pathway that targets a multitude of cytokine and chemokine receptors required for immune recognition, antigen presentation, and adhesion receptors. Similarly, the TNFR2 signaling pathway was also identified by IPA, regulating TNFα activity by antagonizing TNFα-induced apoptosis and the LPS-stimulated MAPK pathway controlling the inflammatory response in macrophage immune cells (58).

### Differentially Expressed Proteins in the Immune Cells of ME/CFS Patients Analyzed by Sequential Window All Theoretical Spectra Mass Spectrometry MS (SWATH-MS)

Proteins from peripheral blood mononuclear cells (PBMC) of ME/CFS patients and age gender matched healthy controls were analyzed to detect differential regulation of specific proteins by Sequential Window all Theoretical Spectra Mass spectrometry MS (SWATH-MS) (33). The fragment ion intensities were aligned to a spectral library constructed from pooled samples from each subject for the identification and quantification of the individual proteins. A total of ~3,000 proteins were quantified reliably by SWATH-MS from triplicate sample measurements.

Nine of the 11 ME/CFS patients segregated from the healthy controls in a Principal Component Analysis (PCA). From a STRING (http://string-db.org, version 11) analysis of the ~100-proteins that were differentially regulated in this ME/CFS group, increased in abundance were immune related proteins (ALOX5, GMFB, GMFG, TPP2, VAMP3, SIPA1, MVP, CD46, IFITM2, MAPKAPK3, VAV1, NCKAP1, SDCBP, CPPED1, DPP7, GCA, UBE2N, ZNRF2, CUL3, PSMB4, PSMD14, PSMB2, SEC61B, SH2DIA), 6 proteasome-related proteins and ~30 mitochondrial proteins, some of which were involved in the electron transport chain and ATP synthesis. Decreased in abundance were immune related proteins (BTN3A1, SLA2, CAPN1, CAB39, METTL7A, PPIA, PRDX3, PRDX6, HEXB, CTSC, SAR1B, VAPA, CPNE3, PAFAH1B2, NME2, HLA-A, HLA-B, CANX, CR1, ERAP2, GP6, C6orf25, GTPBP1, ARPC3, CAPZA1, S100A9, S100A8, VPS4B, VPS4A, RAB34, RAB4A, TMEM, ASH2L), as well as antioxidant proteins, G translation proteins, lysosomal proteins, platelet activation oxygen transport proteins, and Golgi proteins.

This protein dataset reflects a disturbance in the balance of proteins in the peripheral immune cells of patients in the chronic phase of their ME/CFS many years after the onset of their illness, affecting the complexes involved in mitochondrial energy production, and in the mitochondrial regulatory pathway controlling reactive oxygen species. Missailidis et al. demonstrated inefficiency in the production of ATP by Complex V of the mitochondrial electron transport chain, and dysregulated mitochondrial function in immortalized lymphocytes from ME/CFS patients (59). Proteome changes have also been found in mitochondrial metabolism in ME/CFS patients in peripheral immune cells leading to a decreased capacity to provide their cellular energy demands (60). It is now well-recognized that mitochondrial dysfunction, oxidative stress, and neuroinflammation are intrinsically linked (61). Many studies on mitochondria in ME/CFS patients have shown altered membrane potential, condensed cristae and reduced ATP production (59, 62, 63). Mitochondria are responsible for controlling cell death, stimulating inflammatory pathways, and producing ATP involved in purinergic signaling (35, 64). Furthermore, neuroinflammation activates microglia that release reactive oxygen species and reactive nitrogen species that have a deleterious effect on mitochondria (65). This shows that oxidative stress, neuroinflammation and mitochondrial dysfunction are interdependent and potentially in combination could be causative of many of the symptoms seen in ME/CFS.

Recently, Pretorius et al. has discovered microclots, resistant to normal protective fibrinolysis, are an important feature not only of COVID-19 infection but also Long COVID, and they suggested these may interfere with the cells of the body getting sufficient oxygen, thereby providing an explanation for the fatigue (66). Preliminary indications from our comparative proteomic study of ME/CFS and Long COVID patient's immune cells are that the mitochondrial protein imbalance is also present in Long COVID patients and so Long COVID may have an additional restriction on energy production.

Our studies with ME/CFS patients (33) identified 50 dysregulated mitochondrial proteins that created imbalances in the electron transfer molecular complexes leading to the synthesis of ATP as well as in the enzymes involved in mitochondrial metabolism and supported the concept there was a breakdown in molecular homeostasis, and so an analysis of the DNA methylome was undertaken to determine whether changes in expression levels of the transcripts and abundance levels of the proteins were occurring at the DNA level in the methylome.

### A Comparison Between the ME/CFS Patient Cohort and the Healthy Controls Showed Changes in the DNA Methylation Profiles That Reflect Systemic Dysfunctions That Become More Pronounced During a Relapse

Genome scale DNA methylation maps of our ME/CFS patient group and healthy control group were produced using Reduced Representation Bisulphite Sequencing (RRBS) (67, 68). The sequencing data were analyzed utilizing the two analysis platforms, DMAP analysis pipeline (67, 69) to identify differentially methylated fragments, and the MethylKit pipeline (70) was used to quantify methylation differences at individual CpG sites. DMAP identified 76 differentially methylated fragments and Methylkit identified 394 differentially methylated cytosines that indicated both hyper- and hypomethylation was occurring. These clusters identified regulatory regions for 17 protein encoding genes related to immune/inflammatory activity. Analysis of differentially methylated gene bodies (exons/introns) identified 122 unique genes that were affected. Functional pathway enrichment analysis identified 30 pathways associated with these methylome changes. Immune, metabolic, and neurological-related functions were differentially regulated in ME/CFS patients compared to the matched healthy controls. Enriched neurotransmitter and neuropeptide reactome pathways highlighted a disturbed neurological pathophysiology within the patient group.

**Figure 3:**
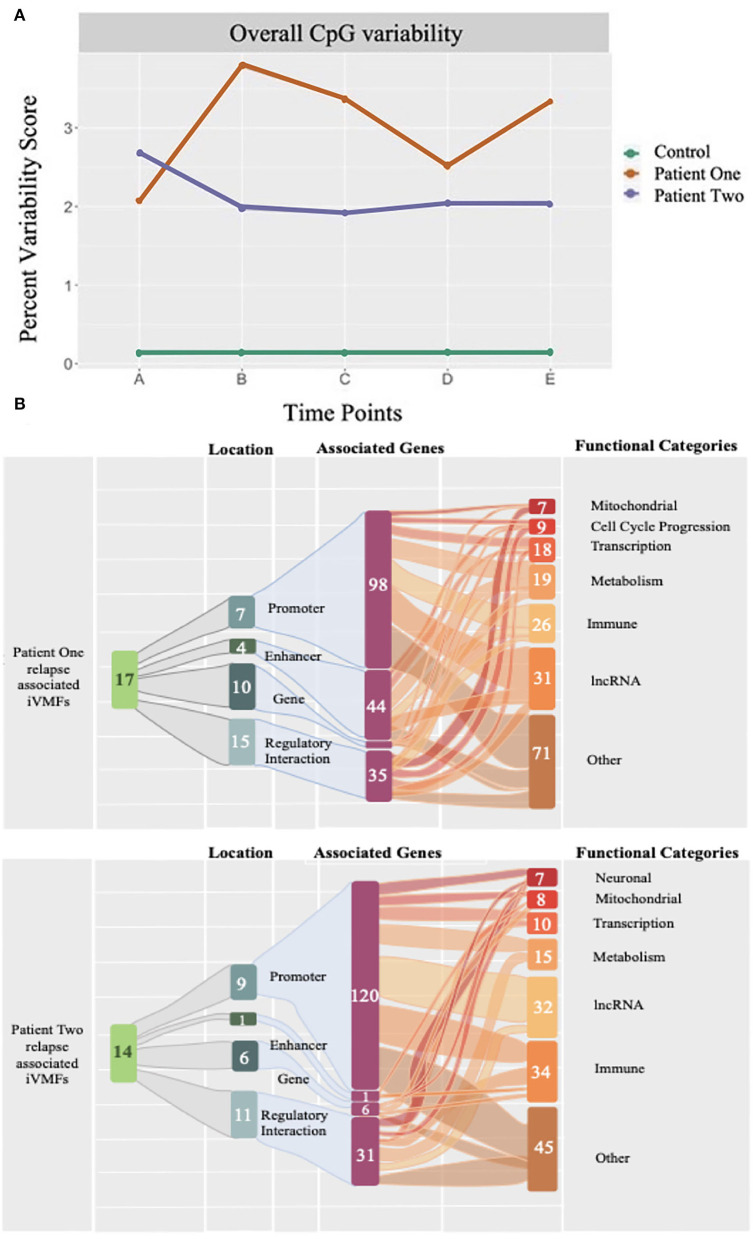
The percent dynamic variability of unique CpG methylations of the epigenetic code in ME/CFS patients and an age gender matched healthy control over a year. DNA methylomes were analyzed at 5 time points for 2 ME/CFS patients and an age/gender matched healthy control. Each point (A-E) represents the statistically significant unique differentially methylated CpG sites at that time point in a longitudinal study lasting 1 year, expressed as a percentage of the total differentially methylated sites determined for that time point (each of A to E). **(A)** The two patients showed a consistent variability of 2–3% over the time course whereas the control variability was constant from sample to sample but 10–20-fold lower (0.15%) than in the patients. **(B)** Each patient suffered a relapse (Time points B, and B, and C, respectively) and in both cases the differentially methylated changes at the relapse time point(s) showed a signature of an enhanced immune response associated with the relapse.

Our most recent study has examined how the immune cell DNA methylome changed in two ME/CFS patients over a year, capturing a serious relapse recovery cycle in each case, together with a control, who remained healthy through the whole period. Reduced representation DNA methylation sequencing profiles were generated from 5 different blood samples for each individual to capture the oncoming relapse and the recovery from it in each patient (49). As shown in Figure 3, comparisons of the DNA methylation changes in each sample over the time course showed both patients had an ongoing methylome instability from sample to sample independent of the relapse, that was 10–20-fold greater than in the control. During the relapse, specific changes were identified in the methylome profile located in regulatory-active regions of the genome that were associated with downstream genes (157 and 127, respectively, for the two patients) indicating significantly disturbed metabolic, immune, and inflammatory functions occurring during the relapse. These findings reinforced the conclusion from our other molecular studies that there was a breakdown in molecular homeostasis in ME/CFS (see Figure 3B).

The above proteome and methylome studies give strong evidence for a dysregulated immune system and widespread chronic systemic inflammation in ME/CFS patients that is sustained long into their illness. These studies support the first part of our hypothesis regarding ME/CFS patients that a chronic systemic inflammation is being maintained well beyond the external stressor event.

## Evidence that Neuroinflammation is Associated with ME/CFS

The important component of the Mackay and Tate proposal was fluctuating neuroinflammation sustaining the ME/CFS illness and central to the occurrence of relapses, affecting the stress center within the PVN of the hypothalamus (32). This could explain not only the widespread pathophysiology of ME/CFS and the still unresolved complex features of ME/CFS, but the sensitivity of patients to even minor normal life stresses of a physical, cognitive, psychological, emotional, and environmental origin. It was consistent with the myriad of symptoms that could be classified as neurological, providing an explanation of why the illness was life-long and did not resolve in most cases. A dysfunctional PVN with a much lower threshold for processing stress signals would be consistent with the ME/CFS patients' sensitivity to even small stresses. This would link neuroinflammation to the well-established involvement of the Hypothalamus/Pituitary/Adrenal (HPA) axis in ME/CFS (71). The hypothalamus connects the nervous system to the endocrine system, regulating both the HPA axis and homeostatic control body temperature, fatigue, sleep, and circadian rhythms- all disturbed in ME/CFS. Mackay wrote an interpretation of these ideas for general health practitioners through the eyes of a sufferer of ME/CFS (72). More recently he has extended these ideas showing they are equally relevant to the large subgroup of the Long COVID patients who are experiencing very similar symptoms to ME/CFS patients (28).

Neuroinflammation implies inflammatory responses within the brain and spinal cord. Microglia, the innate immune cells of the CNS have a central role in mediating this neuroinflammation. The seminal paper that gave evidence of neuroinflammation in ME/CFS in the CNS used the non-invasive imaging technique, Positron Emission Tomography (PET) coupled with Magnetic Resonance Spectroscopy (MRS) together with a radioactive ligand for a translocator protein expressed in activated glial cells as a marker of neuroinflammation (73). ME/CFS patients with varying severity of symptoms were compared with healthy controls to identify the brain regions where neuroinflammation was occurring largely in the limbic system (cingulate cortex, hippocampus, amygdala, and thalamus) a region between the brainstem and the upper regions of the brain. The nearby midbrain and pons region of the brainstem were also potentially affected. Increased binding of the radioactive tracer was ~50–200% higher than in the healthy controls, and an important part of the outcome was a correlation between the severity of the ME/CFS symptoms and the extent of activation of the microglia. While there is yet no published replication of these results the authors report in a personal communication they have successfully replicated their findings with new ligands and they have completed an expanded study that suggested neuroinflammation was occurring at some degree in the hypothalamus (28). A very recent study using the same radioactive ligand puzzlingly found no neuroinflammation in a small cohort of female patients with ME/CFS (74).

The methods available to study possible neuroinflammation in ME/CFS and the difficulties surrounding its measurement, including the method used in both PET/MRS studies have been reviewed in detail (6). The authors also highlight the brain stem as a probable key player in the neuroinflammation's causes and effects. Very recently it has been noted many ME/CFS symptoms can be linked to vital life functions of the brain stem as “the hub relaying information back and forward between the cerebral cortex and various parts of the body” (75). The authors did an integrated evaluation of the brain stem studies carried out to date on ME/CFS patients highlighting structural changes in the white and gray matter, abnormalities of functional connectivity between the brain stem and other regions, indicating neuroinflammation among other mechanisms that might partially explain a significant number of the symptoms of ME/CFS (76). It has also been proposed that persistent brain stem dysfunction occurs in Long COVID as an hypothesis to explain the long-lasting nature of this post-viral illness (77).

Magnetic Resonance Spectroscopy (MRS) has been used to measure brain metabolites (choline, myoinositol, lactate, and N-acetyl aspartate) linked to inflammation and to determine whether brain temperature is elevated in ME/CFS patients and with matched controls. Increased metabolic ratios over control subjects were found that correlated with fatigue in 7/47 brain regions, and increased temperature was observed in several brain regions. It was concluded the findings may indicate neuroinflammation (78). Younger is investigating further a model of low level chronic neuroinflammation in ME/CFS by testing whether peripheral immune cells can enter the brain through a more permeable BBB by introducing radiolabeled immune cells back into patients and observing their migration, having already shown there is no migration in healthy subjects (79).

## Can Neurodegenerative Diseases Give Clues to Neuroinflammation in ME/CFS and Long COVID?

Neuroinflammation is now regarded as a common feature of neurodegenerative disorders. It has been proposed for Alzheimer's disease that “damage signals” such as the appearance of abnormal oligomeric structures of amyloid beta or tau oligomers initiate and sustain glial cell activation that leads to the release of pro-inflammatory cytokines (80). These are endogenous factors that in their abnormal physical state can trigger neuroinflammation. There are no obvious analogies of an aberrant endogenous factor in the CNS in ME/CFS that seem to be at the core of the neurological features of the disease phenotype. What might be useful to promote better understanding of ME/CFS however, is to draw on the information of inflammatory biomarkers that have been found in the cerebrospinal fluid (CSF) for diseases like Multiple sclerosis (MS) (81). Alzheimer's disease (AD) and Parkinson's disease (PD) (80), for example. In the case of MS, the knowledge and rapid assessment of biomarkers has enabled questions of predictive susceptibility, diagnosis, prognosis, disease activity and response to treatment in the different clinical courses of MS. While acknowledging that obtaining spinal fluid is an invasive procedure, it may lead to promising therapeutic options for subgroups of ME/CFS and Long COVID patients. Currently, therapeutic options for ME/CFS are limited, as each promising therapeutic candidate that arises may give some patients benefit, while others experience no effect or have their condition made worse.

## How Does the Systemic Inflammation of the Peripheral System Become Neuroinflammation in the CNS?

**Figure 4:**
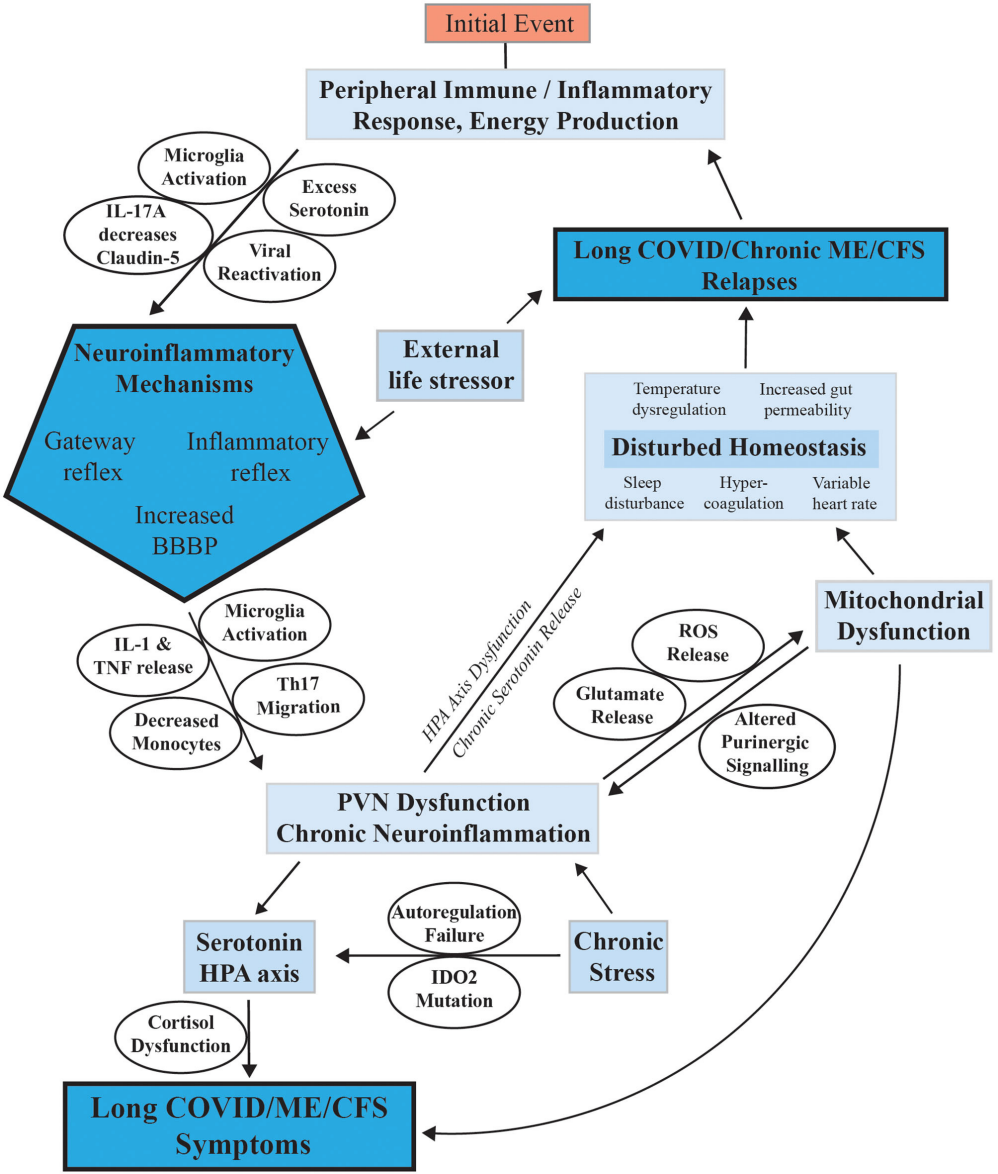
Mechanisms proposed to facilitate the onset of ME/CFS and its progression to a chronic sustained illness with relapse/partial recovery phases. This figure summarizes the mechanisms discussed in the text proposed to have a possible function in sustaining ME/CFS and facilitating relapses.

The question is, through what mechanism does neuroinflammation with manifestation of the ME/CFS symptoms arise often with a gradual onset from the original infection or stress event? We propose here following activation of a systemic immune/inflammatory response to an infection or severe stress event, abnormal transport of signals or molecules into the CNS occurs through neurovascular pathways or a disrupted BBB with intensity that is strong enough to initiate the ME/CFS phenotype firstly with changes in the brain and CNS and then signaling back to the periphery. A summary of the pathways and possible mechanisms is shown in Figure 4.

The following sections explain possible mechanisms for how systemic inflammation of the peripheral system become neuroinflammation in the CNS.

### Inflammatory Reflex

It is possible that the initial systemic inflammatory response in ME/CFS to a viral, bacterial, or other body insult could lead to a neuroinflammatory response *via* the inflammatory reflex as the body sends signals in response to systemic inflammation *via* an afferent/efferent arc through the CNS. The neuroinflammation could then lead to the symptoms commonly associated with ME/CFS. The nervous system is composed of afferent (sensory) and efferent (motor) neurons. The afferent/sensory neurons relay signals from organ systems to the brain where the information is then used to send a response *via* efferent neurons to alter the organ systems function depending on the body's needs (82). The autonomic nervous system controls the body's involuntary systems *via* trillions of afferent nerves responsible for detecting slight changes in temperature, pressure, blood flow and small metabolic changes. This information when received by the brain elicits an efferent response to effect the appropriate change. The sensory neurons detect minute molecular changes within the periphery and detect molecular changes indicative of systemic inflammation.

This recognition of inflammation in the peripheries has been shown to stimulate the HPA axis and create a neuroinflammatory response in the brain as systemic levels of inflammatory mediators can act directly on receptors in the brain suggesting a link between systemic inflammation and neuroinflammation (82–84). In response to infection, mast cells, dendritic cells and macrophages associated with paraganglia have been proposed to release IL-1 that acts on the brain stimulating a neuroinflammatory response (85). Neurons in the CNS have been shown to produce and release cytokines responsible for modulating inflammatory responses such as TNF and IL-1 to peripheral nervous system (PNS) neurons and vice versa in a two-way flow suggesting a pathway between PNS and CNS (82).

Microglia respond to peripheral inflammation by releasing inflammatory cytokines that can lead to a pathological state (86). Although there is little understanding of how peripheral inflammation leads to microglial activation it is possible that the inflammatory reflex plays a key role in this and therefore in the development of neuroinflammation in response to systemic inflammation which could explain many of the symptoms seen in ME/CFS.

### Blood Brain Barrier (BBB)

The blood brain barrier (BBB) is composed of tight junctions formed by endothelial cells that function to prevent substances, cells or molecules from diffusing into the neural tissue of the CNS and doing irreparable damage (87). Blood-brain barrier permeability (BBBP) is controlled by organized functional units made up from microglia, pericytes, astrocytes and the basement membrane (86). It has been proposed that increases in BBBP in patients with ME/CFS lead to many of the symptoms experienced by these patients (87). If any stressor were to lead to dysfunction in the microglia, astrocyte, pericyte and basement membrane functional units this would have a significant impact on the BBBP. Immune cells and neurotoxic molecules would be allowed to enter the brain leading to an immune response in the brain and neuroinflammation, precipitating the symptoms of ME/CFS. To date, many mechanisms have been explored that show increases in BBBP can and do lead to disease.

#### Stress

BBBP can be increased as a *secondary* response to stress. When an external stressor is detected the body releases serotonin. If the initial stress is not alleviated through the PVN this chronic serotonin release can result and cause the serotonin autoregulation pathways to malfunction (88, 89). This in turn leads to excess serotonin release in response to any subsequent stress. Excess serotonin has been shown to lead to increases in BBBP, which in turn leads to neuroinflammation and a subsequent relapse (87). Excess serotonin release and the ensuing neuroinflammation in response to every stress event that a patient encounters in life would explain the ME/CFS relapse-recovery cycle.

#### Systemic Inflammation

Systemic inflammation alone has been shown to lead to increases in BBBP. Intraperitoneal injection of LPS into mice caused microglia to migrate toward cerebral vessels leading to an increase in BBBP (86) explained by microglia's tendency toward phagocytosis in the later stages of inflammation where they engulf astrocyte “endfeet” and cerebral endothelial cells (90). Although the mechanism for chronic microglial activation is unclear this evidence clearly shows that a peripheral event could cause a relapse-recovery cycle due to increased BBBP and chronic microglial activation/reactivation with chronic neuroinflammation as a precursor to all of the symptoms of ME/CFS (91). Astrocytes can also be directly responsible for increasing BBBP. In response to stress, astrocytes secrete IL-17A downregulating the production of the protein claudin-5 produced by microglia (92). Claudin-5 plays a key role in BBB permeability preventing molecules from passing through tight junctions. Without the correct amount of claudin-5 the CNS is vulnerable to molecules that may leak through the BBB. These possible mechanisms for BBB dysfunction could give explanations for the initial neuroinflammation to be established in ME/CFS, the symptoms experienced, and in part facilitate the characteristic relapse-recovery cycle in ME/CFS patients.

#### Chronic Viral Infection or Virus Reactivation

Chronic viral infections in ME/CFS as an effector of ongoing disease has been the subject of much debate. Viruses can remain in the body in latent form however, and they can reactivate with stress. They therefore have the potential to contribute to an immune/inflammatory state that could help maintain the ME/CFS. There is no conclusive evidence of a long term chronic viral infection that maintains ME/CFS, but rather immune dysfunction is caused with the primary infection and that is left as the signature and maintained by other mechanisms (93). If a dormant virus such as the Epstein Barr virus, a common initiating virus in ME/CFS, were to be reactivated in BBB endothelial cells, this would lead to increased BBBP thereby demonstrating a possible mechanism for how viral reactivation could have a role in ME/CFS. Such repeated viral reactivation would result in an inflammatory immune response that could lead to ongoing fluctuating neuroinflammation (87). Over time with each reactivation, the inflammatory response would eliminate the virus-infected cells and the inflammation would subside, however, another relapse could be triggered any time later through the reactivation of virus in other endothelial cells leading to a reactivation-dormancy cycle that would explain the frequent relapse-recovery cycles seen in ME/CFS patients.

### Gateway Reflex

The gateway reflex is a proposed pathway through which immune cells can enter the CNS through a “gateway” in the form of a specific blood vessel (94). A recent study in mice has shown that the L5 cord is a potential site through which pathogenic Th17 cells can move into the CNS *via* L5 dorsal vessels (95). The study showed that in the early stages of encephalomyelitis the chemokine CCL20 attracted pathogenic Th17 cells that subsequently released IL-17 attracting further T-helper cells. It has been shown that increased levels of Th17 levels in the brain contribute to neuroinflammation and neurodegeneration (96). If there were an easy gateway through which CNS entry is possible, this could explain many of the chronic symptoms experienced by ME/CFS patients.

### Gut-Brain Axis

Up until now most research into neurological disease has focused on the CNS itself, however, now it has been indicated that the links between the gut and the brain could play a larger role in the development of many neurological diseases (97, 98). The gut microbiota are vital for maintaining healthy digestion, protecting the body against pathogen invasion, but also for both brain development and protection against degeneration (97, 99). A reduction in microbial diversity (dysbiosis) in the human microbiome is a key feature of many neurological diseases such as multiple sclerosis and Parkinson's (97, 98), with mice studies showing a reduction in object identification, spatial recognition, cortical myelination and BBB function (97, 100). Furthermore, monocyte levels in the CNS were increased in mice following microbiota reconstitution and probiotic use suggesting that dysbiosis can lead to low levels of monocytes that are essential to the immune functionality in the brain leaving the brain vulnerable to neuroinflammation (100). Peripheral tissue damage in the gastrointestinal system can lead to an afferent vagus nerve response that can promote neuroinflammation/degeneration in the brain (83). If an initial external stressor event in an ME/CFS patient were to lead to GI system dysfunction/damage, or dysbiosis in the microbiota from systemic inflammation, then BBB dysfunction, altered immune status in the brain with neuroinflammation could explain many of the symptoms of ME/CFS.

Phytochemicals such as myricitrin, green tea and mulberry that have anti-inflammatory properties have been shown to reduce neurodegenerative pathologies by improving the function and health of the gastrointestinal system (83). Braak (101) suggests that a connection between the enteric nervous system and central nervous system exists through retrograde axonal transport along the vagus nerve. Misfolded aggregates of α-synuclein are proposed to originate in the enteric nervous system and over time migrate into the CNS and brain leading to Parkinson's disease (97, 101, 102). This evidence suggests that there is a strong possibility of a shared link between the gastrointestinal (GI) system and the CNS. Furthermore, while there could be an endogenous factor in ME/CFS that initiates in the GI system and makes its way to the CNS through axonal retrograde leading to and sustaining a neuroinflammatory response, this seems unlikely to be a general effect as ME/CFS has a diversity of initiating causes from viral infection to surgery to severe stress.

## What are the Affected Biological Mechanisms That Result in the ME/CFS-Like State?

### The Hypothalamic-Pituitary-Adrenal (HPA) Axis

Minor stressors such as a temperature change, a glass of wine or a walk around the block often trigger relapse in ME/CFS patients, suggesting that the biological mechanisms responsible for regulating this response to stress are no longer functioning normally. The hypothalamic-pituitary-adrenal (HPA) axis, a neurobiological stress system, is one of the most important mediators of the body's response to stress (71). The HPA axis consists of the paraventricular nucleus (PVN) of the hypothalamus, the pituitary gland and the adrenal glands. In response to an initial stressor or chemical mediator such as serotonin, the PVN will release corticotropin releasing hormone (CRH) and arginine vasopressin (AVP) which act on the anterior lobe of the pituitary gland prompting the production of adrenocorticotropic hormone (ACTH). ACTH travels in the systemic circulation where it acts on the adrenal glands leading to the release of cortisol. Cortisol is important in regulating metabolism, mobilizing glucose to the brain, modulating gluconeogenesis and glycogen synthesis in the liver and induces protein degradation in muscle to facilitate gluconeogenesis. Cortisol also attenuates immune responses by suppressing B and T cell activity (103). The HPA axis is responsible for mediating our inflammatory and immune responses and preventing them from damaging our own body. Hypoactivity/dysfunction of the HPA axis would mean ME/CFS patients' protective response against the body's inflammatory/immune reactions would be diminished (104). In instances where the initial stressor is not alleviated the HPA axis will be repeatedly activated leading to cortisol surges that over time lead to a decrease in cortisol levels. CRH dysfunction, depletion of cortisol, glucocorticoid receptor resistance or impaired cortisol secretion have all been cited as possible causes for this drop off (105). Repetitive injection of inflammatory cytokines over a long period of time have also been shown to lessen the response of the HPA axis showing that chronic systemic or neuroinflammation may lead to HPA axis dysfunction and hypocortisolism (106). It has also been suggested that these low levels of cortisol are the result of upregulated glucocorticoid receptors (GR) and mineralocorticoid receptors (MR) that control cortisol release through negative feedback loops by inhibiting CRH & ACTH release (104). If these receptors are upregulated and the HPA axis is chronically activated over time the negative feedback on the HPA axis may lead to downregulation of the system and a reduction in sensitivity to stress (71). This HPA axis desensitization is known as adrenal fatigue or cortisol dysfunction. Patients with this condition experience symptoms such as myalgia, fatigue, memory loss, brain fog and orthostatic hypotension (105). These symptoms are very similar to those suffered by ME/CFS and Long COVID patients.

A more in-depth mechanism for cortisol dysfunction coined “stress crash” or “adrenal burnout” has been developed. Initially following chronic HPA axis activation, cortisol levels are increased which in turn increases the negative feedback loop on the HPA axis leading to a decrease in cortisol production over time. This increased negative feedback and high levels of cortisol decrease temporarily immune response and production of pro-inflammatory cytokines. Then cortisol levels decrease meaning there is reduced protection by the HPA axis in attenuating these immune/inflammatory responses leaving in this case ME/CFS or Long COVID patients vulnerable to minor stressors that they might encounter in day-to-day life (104). There has also been some discussion around HPA axis sensitivity rather than HPA axis dysfunction. It has been proposed that although there are low levels of ACTH and cortisol in ME/CFS patients their physiological response to low levels of these hormones is the same or even greater than in controls with normal/higher levels of the hormone (88, 107).

The HPA axis can be affected also by stress induced reduced microbial diversity. Increased permeability in the gut wall allows foreign microbes and antigens to cross the epithelial barrier into the surrounding tissue and into the blood and then pass through the blood brain barrier (BBB) eliciting a response from the HPA axis (108). This may then be another contributing factor toward HPA axis dysfunction in ME/CFS as chronic stressors lead to gastrointestinal dysbiosis leading to increased HPA axis activation.

### Serotonin Regulation

In response to an external stressor our body releases serotonin (5-hydroxytryptamine−5HT) from the dorsal raphe nucleus in our brainstem (70). Serotonin has many roles throughout the central nervous system (CNS), modulating other systems in response to stress. Serotonin is also a regulator of the HPA axis through the stimulation of CRH (90). It has been proposed that excess serotonin could in whole or in part be an explanation for the symptoms of ME/CFS (70). The body's serotonin release in response to stress is determined by the CRH receptors 1 and 2 (CRHR's). In periods of high stress CRH is released from neurons in the PVN in large quantities. The high concentration of CRH leads to the internalization of CRHR1 from the membranes of serotonin releasing neurons and replacement by CRHR2 (89). During high periods of stress CRH acts predominantly on CRHR2 leading to raised levels of serotonin. Only at high levels of stress is excess serotonin found in the body, assuming all serotonin regulatory systems are functioning. If the high level of stress continues for a long period of time, excess serotonin can lead to regulatory systems breaking down leading to dysregulation of its production (89).

Serotonin in excess leads to many abnormal symptoms that are indicative of a loss of control over its normal functioning. Symptoms such as dysfunctional muscle contraction through motor neuron inhibition, migraines, sleep disruption, dyspnea, hyperalgesia, and cognitive dysfunction (88, 89). Serotonin can also lead to the release of dopamine and norepinephrine which may explain other physiological changes experienced in ME/CFS with reference to memory, gastrointestinal problems, mood and even blood coagulation (88). Furthermore, it has been shown that the self-oxidation of dopamine to aminochrome can lead to mitochondrial dysfunction which may relate to mitochondrial abnormalities reported in ME/CFS patients (109).

As serotonin can lead to the release of CRH from the PVN of the hypothalamus, chronically high levels of serotonin could lead to chronic reactivation of the HPA axis (110). This is another potential mechanism for HPA axis dysfunction. The combination of both HPA axis dysfunction and excess serotonin would explain most symptoms found in ME/CFS patients; fatigue, pain sensations, mood swings, muscle tightness, insomnia, and cognitive disruption (often called brain fog) and sensory hypersensitivity.

The essential amino acid tryptophan is the precursor to serotonin *via* 5-OH tryptophan. Serotonin, while a beneficial neurotransmitter maintaining mood and other functions, can cause symptoms reminiscent of ME/CFS when present in excess (111).

Tryptophan has a two-enzyme system that act to control its availability for serotonin synthesis through a pathway for production of NAD. The kynurenine pathway, responsible for converting tryptophan into nicotinamide adenine dinucleotide (NAD^+^) (112) has two functionally equivalent enzymes, Indoleamine 2,3-dioxygenase 1 (IDO1) and indoleamine 2,3-dioxygenase 2 (IDO2) responsible for the conversion of tryptophan into kynurenine. Both IDO1 and IDO2 are present at the same point in the pathway and catalyze the same reaction but their activities are dependent on the concentration of tryptophan. IDO1 functions to degrade tryptophan when its concentration is below 200 μM, above which substrate inhibition occurs. IDO2 has low activity at these lower tryptophan concentrations but its activity increases proportionally with the concentration of tryptophan. IDO2 then is like a fail-safe enzyme when tryptophan concentrations become excessive and its metabolism and conversion to kynurenine can still occur. Mutations in the IDO2 gene are quite common in the normal population and it has been hypothesized that having an inactivating mutation in the gene for IDO2 might be a predisposing genetic factor of ME/CFS (113). If tryptophan levels increase to 200 μm where IDO1 stops functioning, and IDO2 is no longer active, then tryptophan levels will continue to increase and be available for serotonin synthesis. The ME/CFS patient is now in a metabolic state with excessive levels of tryptophan and that has been proposed as a disease model for ME/CFS patients (113). If this disturbed tryptophan homeostasis were to occur from an initiating stress, then increase in its levels in susceptible patients with no functional IDO2 could lead to the patient being in “the IDO metabolic trap” (113). We have examined *IDO2* mutations by PCR and sequencing in a cohort of our ME/CFS patients compared with age/gender matched healthy controls. We found 3 of the 5 common mutations were present both as heterozygotes and homozygotes, including those that inactivate or are likely to inactivate the encoded protein, but they were in both control and disease groups (Table 1). We conclude that while the mutation cannot therefore be causative of the ME/CFS it can indeed be a silent susceptibility factor reactivated when ME/CFS illness appears that might help to sustain the illness.

**Table 1:** Examination of the IDO2 gene for mutations in ME/CFS patient and age gender matched healthy controls.

**Amino acid change**	**I140V**	**R248W**	**S252T**	**N257K**	**Y359STOP**
dbSNP ID	rs4736794	rs10109853	rs35212142	rs774492001	rs4503083
Base change	A > G	C > T	T > A	C > G	T > A
Predicted effect	Neutral	Damaging	Neutral/deleterious	Deleterious	Non-sense/inactivated
	**I140V**	**R248W**	**S252T**	**N257K**	**Y359STOP**
Patient 1	+/+	+/−	+/+	+/+	++/+
Patient 2	+/+	+/−	+/+	+/+	+/+
Patient 3	+/+	+/−	+/+	+/+	+/+
Patient 4	+/−	+/+	+/+	+/+	+/−
Patient 5	+/+	+/−	+/+	+/+	+/+
Patient 6	+/+	+/−	+/+	+/+	+/+
Patient 7	+/+	−/−	+/+	+/+	+/+
Patient 8	+/+	−/−	+/+	+/+	+/−
Patient 9	+/+	+/+	+/+	+/+	+/−
Patient 10	+/+	+/+	+/+	+/+	+/+
	**I140V**	**R248W**	**S252T**	**N257K**	**Y359STOP**
Control 1	+/−	+/+	+/+	+/+	+/−
Control 2	+/+	−/−	+/+	+/+	+/+
Control 3	+/+	−/−	+/+	+/+	+/−
Control 4	+/+	+/+	+/+	+/+	+/+
Control 5	+/+	+/−	+/+	+/+	+/−
Control 6	+/+	+/−	+/+	+/+	+/+
Control 7	+/+	−/−	+/+	+/+	+/+
Control 8	+/+	+/+	+/+	+/+	+/+
Control 9	+/+	+/−	+/+	+/+	+/+
Control 10	+/+	+/+	+/+	+/+	+/+

*The upper section shows the common mutations found in the IDO2 gene with the nucleotide and consequential amino acid changes (or creation of a stop signal). The actual genotypes shown in the 10 ME/CFS patients and 10 healthy controls are shown here as “no mutation” (+/+), “heterozygote” (+/–) (dark shade), or “homozygote” (–/–) (lighter shade) in the lower sections*.

Indeed, one study showed heterogeneity of serum tryptophan concentration in ME/CFS patients and therefore availability to the brain and identified provisionally two subgroups of ME/CFS patients, one with normal serotonin, and one with elevated serotonin (114). This then is another possible mechanism additional to the PVN's CRH for increased serotonin levels to contribute to the stress response in ME/CFS patients, with consequential HPA axis activation and eventual dysfunction.

## Testing the Hypothesis

Most studies to date have been carried out on ME/CFS patients in the chronic phase of their illness or at a particular time point of their disease. For example, all our own studies are on patients mostly well into this chronic phase (32–34). To better understand the course of ME/CFS as it relates to this hypothesis, the establishment of study cohorts from the first stages of the illness would be highly beneficial. The patients then individually could be followed though the full course of their illness, from initial stressor event to the development of their ME/CFS and then through its acute and chronic phases (at least for the 75% who progress to this stage) encompassing their incremental improvements and the frequent relapses. This would involve investigating what molecular changes might be sustaining the illness and be preventing recovery from the disease.

### Longitudinal Studies

Studying ME/CFS from the initial viral infection or stress event is a challenge as there are currently no tools that predict who will go on to develop the disease maybe weeks or even several months later, and as well the disease is diagnosed only formally after 6 months of ongoing ME/CFS-like unresolved symptoms. By contrast Long COVID would be easier to study in its full course from the point of viral infection as there are so many people infected with SARS-CoV-2 in the pandemic and the frequency of those developing Long COVID is high, so a post-viral cohort would more easily be obtained. However, it should be noted a significant proportion of cases of ME/CFS are derived from Epstein Barr virus infections at quite a high frequency also, so establishing a cohort of those infected with this virus from the time of infection could lead onto a study cohort for ME/CFS. These patients could be studied throughout the course of their illness to map the molecular and physiological changes that are occurring. This would establish a solid platform to understand the disease and for testing hypotheses like the one proposed here.

### Brain and CNS Imaging Technologies

Neuroinflammation is a central feature of our hypothesis and imaging technologies are critical for its development and further testing. The resolution of MRS currently allows conclusions on the global changes occurring in the brain in ME/CFS (73), but advances in resolution would greatly enhance fine mapping of the regions and neuron clusters of the brain that may be structurally or functionally affected in ME/CFS. Since our hypothesis encompasses fluctuating neuroinflammation for both ME/CFS and Long COVID (28, 29) as a determinant of the frequent relapses in ME/CFS it would be ideal if these technologies became routine, and then patients could be evaluated regularly throughout the course of their illness. Since ME/CFS is not neurodegenerative and, thereby potentially reversible, gaining evidence of waxing and waning neuroinflammation would be critical to the importance of the hypothesis if it can be linked to improvement or deterioration in functionality and symptoms in ME/CFS. Recent advances in the resolution of structural changes in the brain stem in ME/CFS give promise that advanced neuroimaging tools will become available for other brain regions and continue to contribute to understanding the brain function and the neurological symptoms of ME/CFS (6). These advances will provide essential information to evaluate our hypothesis. PET imaging has a need for more reliable quantification of neuroinflammation (115).

To investigate whether immune cells activated in a systemic inflammatory response are entering the CNS through the “gateway reflex” discussed above in ME/CFS patients, imaging from PET coupled with MRS can be applied to identify specific cell surface markers on T cells (116, 117). If there were evidence for aggregation of T cells around a specific area of the spinal cord or visual representation of a migration of T cells from the systemic circulation into the CNS this would provide evidence for the gateway reflex as a contributor to the pathophysiology of ME/CFS.

Increases in permeability of the BBB (BBBP) as discussed above would allow immune cells, metabolites, or cytokines to migrate into the CNS and precipitate the proposed neuroinflammation. Dynamic contrast-enhanced magnetic resonance imaging (DCEMRI) or dynamic perfusion CT (PCT) are imaging techniques that could be used to take a quantitative measure of BBBP (118, 119). Evidence of increased BBBP would add support for our hypothesis.

### Cerebrospinal Fluid (CSF) Sampling as a Tool for Following the Progression of ME/CFS

Extensive sampling of CSF in ME/CFS patients has been avoided to date as it is an invasive procedure, and the patients under evaluation are often quite unwell. However, if we are to understand the longitudinal course of the ME/CFS and the molecular changes occurring in the brain, then regularly evaluating the CSF and its pool of potential biomarkers to detect dynamic changes could be a valuable tool. It may provide significant insight into the current hypothesis. CSF sampling has been used in MS to evaluate the course of the disease, treatment options and for general management strategies (80). This approach could be linked to therapeutic developments aimed at suppressing the neuroinflammation and the debilitating neurological symptoms of the disease.

### Inflammatory Reflex

Since neuroinflammation is at the center of our hypothesis for ME/CFS, the inbuilt inflammatory reflex within the body could be targeted therapeutically to mitigate the neuroinflammatory response. This has been proposed by Tracey with a small molecule such as CNI-1493 used to stimulate anti-inflammatory mechanisms in the brain (82).

### Excessive Serotonin Production

A significant change in tryptophan concentration in the blood from disturbed homeostasis will be mirrored by a change in serotonin synthesis, with tryptophan hydroxylase the rate limiting enzyme of the serotonin-biosynthetic pathway. Entry of tryptophan into the brain is limited by the concentration of five other amino acids (valine, leucine, isoleucine, phenylalanine, and tyrosine) known as the competing amino acids (CAA) (114). To test whether excess serotonin production could be linked to the pathophysiology of ME/CFS, in one study it was demonstrated that some ME/CFS patients in comparison to controls had higher tryptophan to CAA ratios but similar levels of CAA (114). If this study can be replicated with other cohorts it would provide evidence that high systemic tryptophan levels that resulted in increased serotonin production in the brain were likely contributing to the neurological symptoms of ME/CFS patients.

## What Determines Who Develops the Post-Viral/Stress Fatigue Syndromes?

A pressing question about the development of post-viral/stress fatigue syndromes is what determines those in the community who develop these long-term conditions in contrast to the majority of people who go through a typical acute response and then a healing cycle. For example, up to 1 in 10 who contract Epstein Barr virus-mediated glandular fever will go on to develop ME/CFS, and at a lower frequency after a major stress event like surgery or after exposure to agricultural chemicals like organophosphates. A range of frequencies from as high as 1 in 2 to as low as 1 in 10 have been published for those who develop Long COVID after SARS-CoV-2 infection (120). Now data are emerging that the current vaccination campaign against this virus is leading to severe ongoing relapse in ME/CFS patients of their fatigue syndrome at a frequency of 1 in 4 in New Zealand (121) but also in healthy individuals at a much lower frequency. All these responses relate to how each individual's immune system reacts to the external stressor—and that is the common factor in the development of the illnesses. Some members of our community have an immune/inflammatory reaction above a safe threshold irrespective of the stressor that does not allow them to transition into the healing resolving response, but rather they transition into a cycling ongoing condition fueled and maintained by the typical everyday stresses of living.

## Data Availability Statement

Datasets generated and analysed for Figure 3 of this study can be found in the GEO database NCBI (GSE166592), see also (49).

## Ethics Statement

The studies involving human participants were reviewed and approved by Health and Disability Ethics Committee of New Zealand - ethics approval 17/STH/188. The patients/participants provided their written informed consent to participate in this study.

## Author Contributions

WT conceived and designed the hypothesis and wrote the paper. MW researched the ideas and helped develop the hypothesis and write the paper. ES did experimental molecular studies underpinning part of the hypothesis, contributed to the writing, and critiqued the manuscript. AH did experimental molecular studies underpinning the hypothesis and critiqued the manuscript. KP helped analyze data, prepared diagrams, and critiqued the manuscript. CE and AB analyzed ID02 gene mutations in ME/CFS patients and healthy controls and critiqued the manuscript. AC provided fundamental expertise and supervision of the DNA methylome studies and critiqued the manuscript. All authors contributed to the article and approved the submitted version.

## Funding

The project was supported by grants from the Healthcare Otago Charitable Trust, the Associated New Zealand Myalgic Encephalomyelitis Society (ANZMES), and generous private donations from ME/CFS families.

## Conflict of Interest

The authors declare that the research was conducted in the absence of any commercial or financial relationships that could be construed as a potential conflict of interest.

## Publisher's Note

All claims expressed in this article are solely those of the authors and do not necessarily represent those of their affiliated organizations, or those of the publisher, the editors and the reviewers. Any product that may be evaluated in this article, or claim that may be made by its manufacturer, is not guaranteed or endorsed by the publisher.
